# Effects and mechanisms of docosahexaenoic acid on the generation of angiopoietin-2 by rat brain microvascular endothelial cells under an oxygen- and glucose-deprivation environment

**DOI:** 10.1186/s40064-016-3067-7

**Published:** 2016-09-09

**Authors:** Xiaobo Chen, Qiang Wang, Leyun Zhan, Aihua Shu

**Affiliations:** Department of Anesthesiology, Three Gorges University People’s Hospital, The First People’s Hospital of Yichang, No. 2 Jiefang Road, Yichang, 443000 Hubei China

**Keywords:** DHA, OGD environment, COX-2, Ang-2

## Abstract

**Objective:**

The aim of this study was to investigate the effects of docosahexaenoic acid (DHA) on the generation of angiopoietin-2 (Ang-2) by rat brain microvascular endothelial cells under an oxygen- and glucose-deprivation environment (OGD), and its relationship, if any, with cyclooxygenase 2 (COX-2) expression.

**Methods:**

Annexin V and propidium iodide apoptosis assay was used to detect apoptosis. Enzyme linked immunosorbent assay was used to detect Ang-2, vascular endothelial growth factor (VEGF), prostaglandin E2 (PGE2), and prostaglandin I2 (PGI2) content. Reverse transcription polymerase chain reaction (RT-PCR) was used to detect Ang-2 and VEGF mRNA expression. Western blot was used to detect expression of COX-2 protein.

**Results:**

DHA reduced the apoptosis rate (*P* = 0.026) and decreased the secretion of Ang-2, VEGF, PGE2, and PGI2 (*P* = 0.006, *P* = 0.000, *P* = 0.002, *P* = 0.004 respectively). The relative expression of *Ang2* and *Vegf* mRNA, as well as COX-2 expression, also decreased (*P* = 0.000, *P* = 0.005, *P* = 0.007 respectively). These effects were antagonized by GW9662 (peroxisome proliferator-activated receptor-γ antagonist). COX-2 protein expression levels were positively correlated with *Ang2* and *Vegf* mRNA expression levels (γ = 0.69, *P* = 0.038 and γ = 0.76, *P* = 0.032, respectively). *Ang*-*2* and *VEGF* mRNA levels were positively correlated with Ang-2 (γ = 0.84, *P* = 0.012) and VEGF (γ = 0.71, *P* = 0.036) secretion levels respectively.

**Conclusion:**

DHA reduced apoptosis induced by an OGD environment, thus decreasing Ang-2 and VEGF synthesis. This phenomenon was associated with a decrease in COX-2 protein expression, PGE2 and PGI2 secretion, and generation regulation via intracellular transcriptional pathways.

## Background

Studies have confirmed that the angiopoietin (Ang)-Tie2 signaling pathway is related with functional recovery and prognosis after brain injury (Wang et al. 2015; Chittiboina et al. 2013; Fischer et al. 2011). Ang-1 is constitutively produced by pericytes and can be induced in several perivascular cell types. Ang-2 is released from the Weibel-Palade bodies of brain microvascular endothelial cells (BMVEC) in response to hypoxia or inflammatory stimuli, and competes with Ang-1 for Tie-2 receptor binding (Oike et al. 2004). In the early stages of brain injury, Ang-2 expression rapidly increases, whereas Ang-1 amounts decrease. The subsequent Ang-1/Ang-2 imbalance leads to impaired Tie-2 activation and reduction of Tie-2 auto-phosphorylation (Kawamura et al. 2014). Tie-2 phosphorylation can increase the expression of phosphoinositide 3-kinase (PI3 K)-Akt signaling pathway proteins, which has been closely linked to cell survival, as well as the expression of endothelial composite materials and endothelial cell gap junction stabilization (Hill et al. 2014; Yu et al. 2012; Ruan and Kazlauskas 2013). Therefore, during early ischemia, the intervention and regulation of the Ang/Tie2 signaling pathway by increasing Ang-1 or decreasing Ang-2 levels may be an effective strategy for protecting the brain.

Moreover, the mechanistic functioning of Ang-2 on the endothelial cell appears to reflect coordinate signaling between Ang-1 abundance, VEGF receptor occupancy and signaling, and other signaling systems. Ang1 opposes the VEGF-induced de-differentiation of BMVEC (Shen et al. 2011), but its effects on BMVEC early after ischemia are impeded by a rapid release of Ang2. VEGF may play a role in modulating the function of Ang-2. Therefore, it is clear that any evaluation of Ang-2/Ang-1 participation in injury should also consider how VEGF levels are affected (Lobov et al. 2002). Increasing evidence strongly supports the role of cyclooxygenase-2 (COX-2) and its metabolic product prostaglandin (PG) E2 as regulators of angiogenesis. Under hypoxic conditions, Ang-2 levels have been closely linked to the expression levels of COX-2, PGE2, and PGI2. Inhibiting COX-2 or reducing PGE2 and PGI2 expression can significantly downregulate Ang-2 (Iniguez et al. 2003; Pichiule et al. 2004).

In the past decade, the health benefits of unsaturated fatty acids, mainly docosapentaenoic acid and docosahexaenoic acid (DHA), have attracted increasing research interest. Eicosapentaenoic acid (EPA) and DHA, which belong to the n-3 polyunsaturated fatty acid (PUFA) family, are the main components of deep-sea fish oil. Fish oil, a type of peroxisome proliferator-activated receptor (PPAR) γ natural ligand, has been reported to slow the progress of kidney disease. A diet rich in n-3 PUFAs, like EPA and DHA, has been associated with reduced triacylglycerol (TG) levels and pulmonary disease, as well as increased anti-inflammatory activity and a decreased risk of cardiovascular disease and mortality (Moertl et al. 2011). Moreover, the present study showed that treatment with unsaturated fatty acids can exert neuroprotective effects in both acute and chronic brain injury (Mills et al. 2011a, b). Although three PPAR receptor subtypes (α, β/δ, γ) are involved in the pathophysiology of brain damage, PPAR-γ was the most closely linked to brain injury, and is currently the most thoroughly studied receptor (Benedusi et al. 2012). Anti-apoptotic, anti-inflammatory, and anti-oxidant were the main mechanisms of action (Figueroa et al. 2012; Quartu et al. 2012). However, very few studies regarding the relationship between the mechanisms of DHA brain protection and the Ang/Tie2 signaling pathway are available at the present. Therefore, we hypothesized that DHA may decrease Ang-2 expression by activating PPAR-γ and inhibiting COX-2. This study investigated the effects and mechanisms of DHA on the generation of Ang-2 in BMVECs under an oxygen- and glucose-deprivation (OGD) environment, providing a scientific basis for future clinical application.

## Methods

### Isolation and culture of BMVECs

Male Sprague–Dawley (SD) rats (2–3 weeks old, 40–60 g) were provided by the Science and Technology Laboratory Animal of Huazhong University. BMVECs was isolated, purified, and cultured according a previously described method (Abbott et al. 2012). BMVECs was identified by immunocytochemistry using an anti-VIII factor-related antigen antibody. More than 95 % of 3rd generation cells were BMVECs, which were used in experiments. This study was carried out in strict accordance with the recommendations in the Guide for the Care and Use of Laboratory Animals of the National Institutes of Health. The animal use protocol has been reviewed and approved by the Institutional Animal Care and Use Committee (IACUC) of the First People’s Hospital of Yichang.

### Preparation and organization of OGD models

The cells were divided into groups based on treatment: control group, OGD group, OGD + 10 μM DHA group, OGD + 40 μM DHA group, OGD + 10 μM DHA + GW9662 group, and OGD + 40 μM DHA + GW9662 group. Third generation BMVECs were digested into single cell suspensions, cell density was adjusted to 1 × 10^5^/mL, and seeded in 24-well plates (six parallel wells for each set). Cells were then cultured for 24 h under 5 % CO_2_ condition for synchronization. The original culture of OGD and DHA pretreatment groups were then replaced with sugar-free, serum-free DMEM culture media. At this point, the two concentrations of DHA was added to the DHA pretreatment groups, and 5 μM GW9662 was added to the OGD + 10 μM DHA + GW9662 and OGD + 40 μM DHA + GW9662 groups. All cells were then cultured for 1 h and then placed into the hypoxic incubator (94 % N_2_, 5 % CO_2_, 1 % O_2_) for 24 h. DMEM media with serum, 5 mM/L glucose and 1.25 mM pyruvate was added to the control group, which was cultured under 5 % CO_2_ and 95 % air conditions. After culturing for 24 h, the supernatants from each group were collected and stored at −20 °C for enzyme linked immunosorbent assay (ELISA) analysis. Ang-2, vascular endothelial growth factor (VEGF), PGE2, and PGI2 content in the supernatants were detected using ELISA kits in accordance with manufacturer instructions. The cells were harvested and pelleted for reverse transcription polymerase chain reaction (RT-PCR) and Western blot.

### Apoptosis detection by flow cytometry

Apoptosis detection was performed in accordance with the manufacturer’s instructions using the Annexin V & propidium iodide (PI) apoptosis detection kit (Nanjing KGI Biology, China). Briefly, 5 μl Annexin V, 5 μl PI, and 500 μl Binding Buffer was added to each sample and mixed at room temperature while protected from light. Reaction time was 5–15 min, and the negative control was normal cells without addition of Annexin V and PI. The first positive control was the solvent group with the most obvious apoptosis, with only 5 μl Annexin V single standard. The second positive control was the solvent group with the most obvious apoptosis, with only 5 μl PI single standard. Detection was performed using flow cytometry.

### ELISA

Specific Ang-2, VEGF, PGE2, and PGI2 ELISA detection kits were purchased from Eli Reiter Biotechnology Co. Ltd. (Wuhan, China). Blanks, standard controls, and test samples were added to microtiter plates. Blanks, controls, and samples were diluted to 100 μl. Labeled antibodies and the biotin were co-incubated with the test samples. After washing, the avidin HRP-labeled biotin was added to facilitate binding with the immune complexes. After several incubation and washing cycles, the unbound conjugate was gradually removed. The added substrate reacted and caused a visible color reaction, which showed a proportional relationship with the concentrations present in the samples. The optical density (OD) values of the samples were detected using an automatic biochemical analyzer. Standard curves were plotted according to standard OD values, and test sample concentrations were calculated from the standard curve.

### RT-PCR

Cells were collected and total RNA was extracted using Trizol (TAKARA, Dalian, China) and detected using real-time PCR. The expression of the β-actin gene was used as an internal reference control. RT-PCR reaction conditions were established using conventional methods. The sequences of the *Ang2* primers were: upstream primer, 5′-AGCCAGTCTCCCTTCCAG-3′, downstream primer, 5′-AGGCAAGCCATTCTCACA-3′, amplicon 123 bp; *Vegf* primer sequences were: upstream primer, 5′-ATCAAGGCCGTCTGTGGT-3′, downstream primer, 5′-GGTTTCGGAGGTCAGTGTT-3′, amplicon 287 bp; internal reference β-actin primer sequences: upstream primer, 5′-CACGATGGAGGGGCCGGACTCATC-3, downstream primer, 5′-TAAAGACCTCTATGCCAACACAGT-3′, amplicon 240 bp. Reaction system: reaction volume of 20 μl, containing 4 μl cDNA, 0.4 μl primers. The reaction conditions were 50 °C for 2 min, 95 °C for 10 min, 40 cycles of 95 °C for 30 s and 60 °C for 30 s. Fluorescence signals were collected during the annealing stage, and the results were analyzed using real-time quantitative analysis software for the ViiA7 real-time PCR instrument.

### Western blot

Appropriate RIPA lysis buffer (Pik Days, Shanghai, China) was used to lyse cells, which were then centrifuged to obtain and extract the supernatant. Protein concentrations for each sample were then measured, the total protein was sampling for electrophoresis. Electric water bath blotting was used to transfer proteins to PVDF membranes (Millipore, Germany). The corresponding anti-blocking solution was used to dilute primary antibodies. The membranes were immersed in rabbit anti-mouse COX-2 antibody (Santa Cruz, Shanghai, China) incubation media and incubated overnight at 4 °C. PVDF membranes were washed thoroughly with TBST 5–6 times, 5 min each. Blocking solution was used to dilute corresponding HRP-labeled secondary antibodies (Wuhan Boster Biological Engineering Co., Ltd., Wuhan, China). Membranes were then immersed in secondary antibody incubation media, incubated on a shaking bed at room temperature for 2 h. PVDF membranes were then sufficiently washed with TBST for 5-6 times, 5 min each. An appropriate amount of ECL substrate solution (Thermo, Shanghai, China) was added to each membrane and incubated for several minutes. After fluorescence bands were visible, developing and fixing solutions were added in turn. Each experiment was repeated three times. The densitometry of the targeted protein COX-2 and reference bands were analyzed using a biological electrophoresis image analysis system, and the relative expression of the targeted protein was obtained by comparing the densitometry of the targeted bands and internal references for the same sample.

### Statistical analysis

SPSS v.13.0 software analysis was used for data analysis. Data were expressed as mean ± standard deviation ($\overline{x}$ ± s). One-way ANOVA (multiple comparison among group) and SNK (pairwise comparison between groups) tests were used to compare differences between groups.

## Results

### Apoptosis in BMVECs

**Fig. 1:**
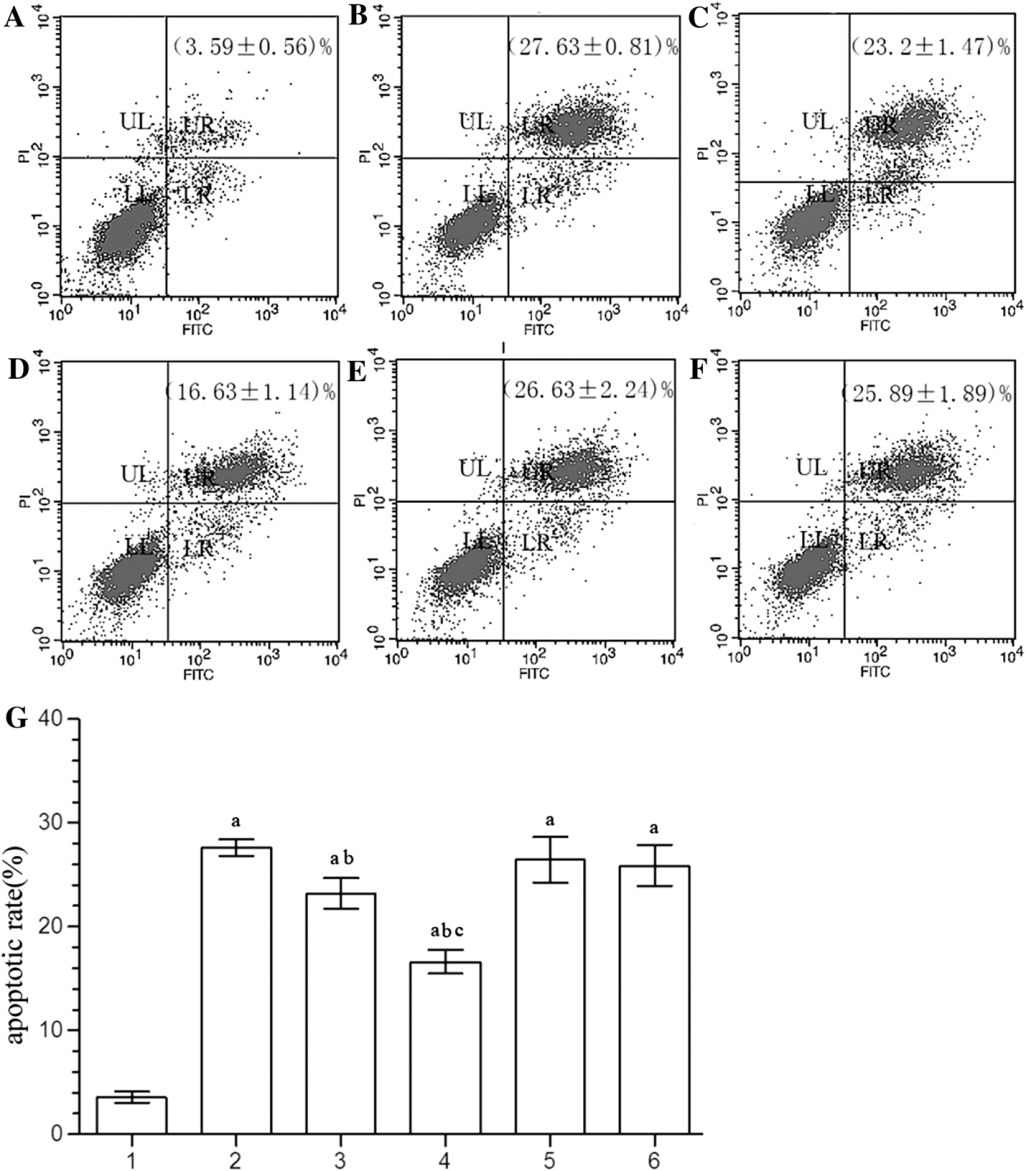
**A**, **B**, **C**, **D**, **E**, **F** Scatter plot of apoptosis. **A** control group, **B** OGD, **C** OGD + 10 μM DHA, **D** OGD + 40 μM DHA, **E** OGD + 10 μM DHA + GW9662, **F** OGD + 40 μM DHA + GW9662, **G** histogram of apoptosis.* 1* control group,* 2* OGD,* 3* OGD + 10 μM DHA,* 4* OGD + 40 μM DHA,* 5* OGD + 10 μM DHA + GW9662,* 6* OGD + 40 μM DHA + GW9662. Compared with the control group, ^a^ *P* < 0.01; compared with OGD group, ^b^ *P* < 0.01; compared with 10 μM DHA pretreated group, ^c^ *P* < 0.01

Apoptosis in BMVECs of the OGD, OGD + 10 μM DHA, OGD + 40 μM DHA, OGD + 10 μM DHA + GW9662, and OGD + 40 μM DHA + GW9662 groups increased significantly compared with the control group (*P* < 0.01). Apoptosis of the OGD + 10 μM DHA group and OGD + 40 μM DHA groups was significantly lower compared to the OGD group (*P* < 0.05). The OGD + 10 μM DHA + GW9662 and OGD + 40 μM DHA + GW9662 group showed no significant differences in apoptosis. The OGD + 40 μM DHA group had significant differences in apoptosis compared with the OGD + 10 μM DHA group (*P* < 0.05; Fig. 1).

### Secretion of Ang-2, VEGF, PGE2, and PGI2

**Fig. 2:**
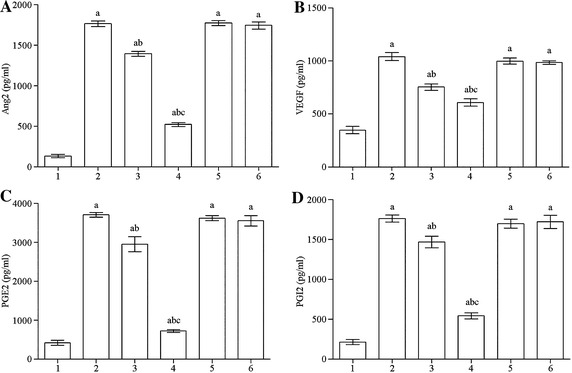
**A** Ang2 secretion of each group. *1* control group, *2* OGD, *3* OGD + 10 μM DHA, *4* OGD + 40 μM DHA, *5* OGD + 10 μM DHA + GW9662, *6* OGD + 40 μM DHA + GW9662. Compared with the control group, ^a^ *P* < 0.01; compared with OGD group, ^b^ *P* < 0.01; compared with the 10 μM DHA pretreated group, ^c^ *P* < 0.01. **B** VEGF secretion in each group. *1* control group, *2* OGD, *3* OGD + 10 μM DHA, *4* OGD + 40 μM DHA, *5* OGD + 10 μM DHA + GW9662, *6* OGD + 40 μM DHA + GW9662. Compared with the control group, ^a^ *P* < 0.01; compared with OGD group, ^b^ *P* < 0.01; compared with the 10 μM DHA pretreated group, ^c^ *P* < 0.01. **C** PGE2 secretion of each group. *1* control group, *2* OGD, *3* OGD + 10 μM DHA, *4* OGD + 40 μM DHA, *5* OGD + 10 μM DHA + GW9662, *6* OGD + 40 μM DHA + GW9662. Compared with the control group, ^a^ *P* < 0.01; compared with OGD group, ^b^ *P* < 0.01; compared with the 10 μM DHA pretreated group, ^c^ *P* < 0.01. **D** PGI2 secretion levels in each group. *1* control group, *2* OGD, *3* OGD + 10 μM DHA, *4* OGD + 40 μM DHA, *5* OGD + 10 μM DHA + GW9662, *6* OGD + 40 μM DHA + GW9662. Compared with the control group, ^a^ *P* < 0.01; compared with OGD group, ^b^ *P* < 0.01; compared with the 10 μM DHA pretreated group, ^c^ *P* < 0.01

Compared with the normal control group, Ang-2, VEGF, PGE2, and PGI2 secretion levels of the OGD, OGD +10 μM DHA, OGD + 40 μM DHA, OGD + 10 μM DHA GW9662, and OGD + 40 μM DHA + GW9662 groups were significantly increased (*P* < 0.01). Compared with the OGD group, Ang-2, VEGF, PGE2, and PGI2 secretion of the OGD + 10 μM DHA and OGD + 40 μM DHA groups decreased significantly (*P* < 0.01), while the OGD + 10 μM DHA + GW9662 and OGD + 40 μM DHA + GW9662 groups showed no significant difference. Compared with the OGD + 10 μM DHA group, OGD + 40 μM DHA group had a significant difference (*P* < 0.01; Fig. 2A–D).

### *Ang2* and *Vegf* mRNA expression

**Fig. 3:**
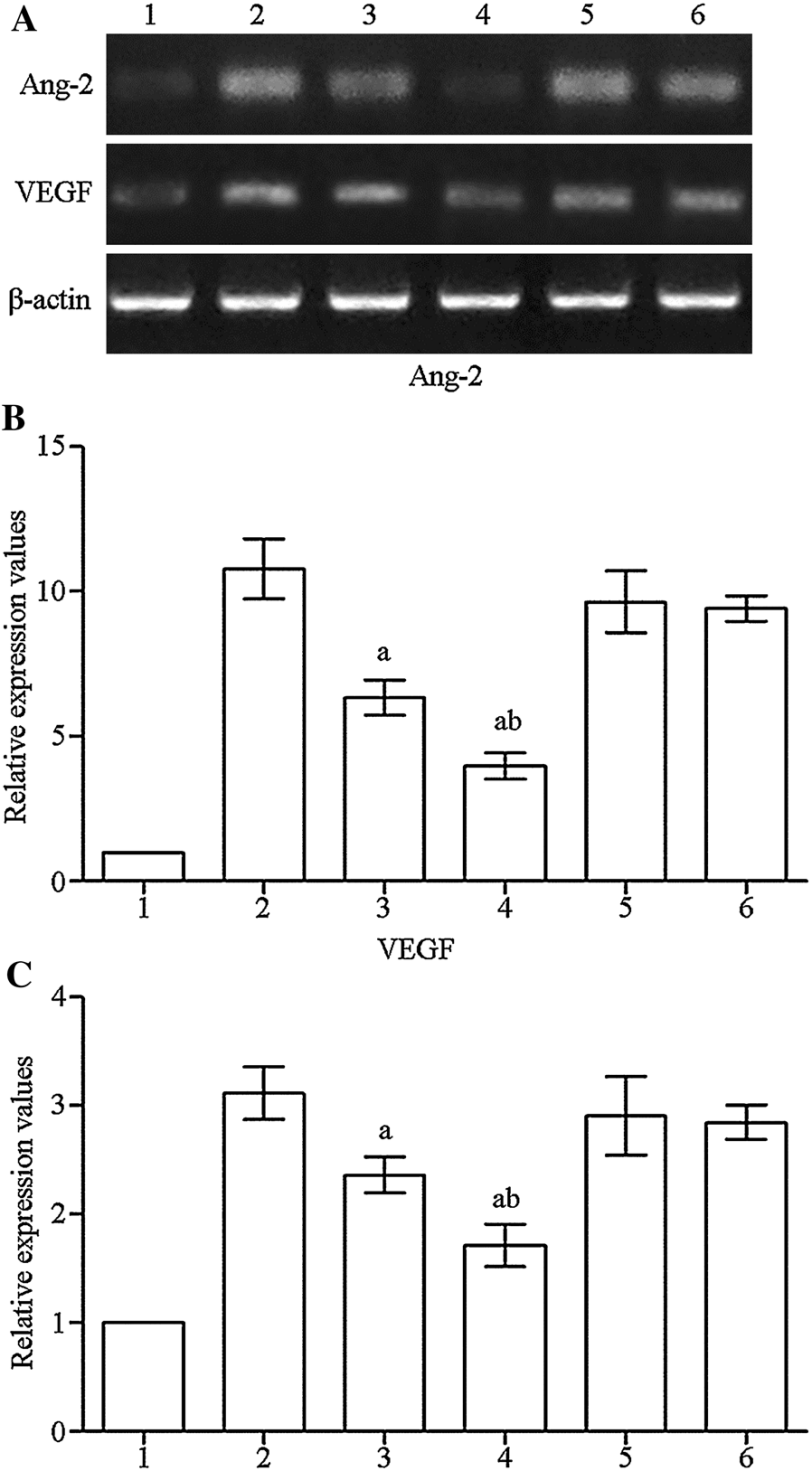
**A** RT-PCR strip chart for Ang2 and VEGF. *1* control group, *2* OGD, *3* OGD + 10 μM DHA, *4* OGD + 40 μM DHA, *5* OGD + 10 μM DHA + GW9662, *6* OGD + 40 μM DHA + GW9662. **B** RT-PCR histogram for Ang2. *1* control group, *2* OGD, *3* OGD + 10 μM DHA, *4* OGD + 40 μM DHA, *5* OGD + 10 μM DHA + GW9662, *6* OGD + 40 μM DHA + GW9662. Compared with OGD group, ^b^ *P* < 0.01; compared with the 10 μM DHA pretreated group, ^c^ *P* < 0.01. **C** RT-PCR histogram for VEGF. *1* control group, *2* OGD, *3* OGD + 10 μM DHA, *4* OGD + 40 μM DHA, *5* OGD + 10 μM DHA + GW9662, *6* OGD + 40 μM DHA + GW9662. Compared with OGD group, ^b^ *P* < 0.01; compared with the 10 μM DHA pretreated group, ^c^ *P* < 0.01

2^−ΔΔCt^ quantitative analysis was used. *Ang2* and *Vegf* mRNA expression in OGD, OGD + 10 μM DHA, OGD + 40 μM DHA, OGD + 10 μM DHA + GW9662, and OGD + 40 μM DHA + GW9662 pretreatment groups were 10.8-, 6.4-, 3.9-, 9.6-, 9.4-fold greater (*P* < 0.01) and 3.1-, 2.3-, 1.7-, 2.9-, 2.8-times greater (*P* < 0.01) than the control group respectively. Compared with the OGD group, *Ang2* and *Vegf* mRNA expression in the 10 μM DHA and 40 μM DHA pretreatment groups was significantly reduced, while the OGD + 10 μM DHA + GW9662 and OGD + 40 μM DHA + GW9662 group showed no significant difference (*P* > 0.05). Compared with the OGD + 10 μM DHA group, the OGD + 40 μM DHA group had significant difference (*P* < 0.01; Fig. 3).

### Expression of COX-2 protein

**Fig. 4:**
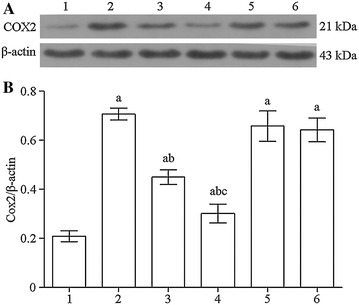
**A** Western blot strip chart for COX2. *1* control group, *2* OGD, *3* OGD + 10 μM DHA, *4* OGD + 40 μM DHA, *5* OGD + 10 μM DHA + GW9662, *6* OGD + 40 μM DHA + GW9662. **B** Western blot histogram for COX2. *1* control group, *2* OGD, *3* OGD + 10 μM DHA, *4* OGD + 40 μM DHA, *5* OGD + 10 μM DHA + GW9662, *6* OGD + 40 μM DHA + GW9662. Compared with the control group, ^a^ *P* < 0.01; compared with OGD group, ^b^ *P* < 0.01; compared with the 10 μM DHA pretreated group, ^c^ *P* < 0.01

Western blotting assay showed that, compared with the control group, COX-2 protein expression in the OGD, OGD + 10 μM DHA, OGD + 40 μM DHA, OGD + 10 μM DHA + GW9662, and OGD + 40 μM DHA + GW9662 groups significantly increased (*P* < 0.01). Compared with the OGD group, COX-2 expression of the 10 μM DHA and 40 μM DHA pretreatment groups decreased significantly (*P* < 0.01), while OGD + 10 μM DHA + GW9662 and OGD + 40 μM DHA + GW9662 groups showed no significant difference. The COX-2 protein expression of the 40 μM DHA pretreatment group was significantly lower than the 10 μM DHA pretreatment group (*P* < 0.01; Fig. 4).

### Correlation between COX-2 protein expression with *Ang2* and *Vegf* mRNA

COX-2 protein expression was positively correlated with *Ang2* and *Vegf* mRNA levels; the correlation coefficients were 0.69 and 0.76 (*P* < 0.05) respectively. *Ang2* and *Vegf* mRNA and Ang2 and VEGF secretion levels were positively correlated, with the correlation coefficients being 0.84 and 0.71 (*P* < 0.05) respectively.

## Discussion

It has been demonstrated extensively in the literature that Ang-2 levels were elevated in the cerebrospinal fluid of patients with brain damage, whereas Ang-1 levels were reduced. Alterations in the cerebrospinal fluid levels of these markers may provide a new clinical index to predict disease severity, or represent different mechanisms of brain injury (Kurosaka et al. 2010; Chen et al. 2010). The interaction between Ang-1 and Ang-2 allowed researchers to re-examine how to perform angiopoietin system targeting therapy for patients with severe brain injury to improve blood–brain barrier and reduce brain edema. For some chronic diseases such as diabetes, the use of DHA can effectively reduce plasma levels of Ang-2. This mechanism may have some beneficial effects in the treatment of ischemic brain injury (Nomura et al. 2009). Our results showed that DHA can reduce apoptosis of BMVECs and generation of Ang-2 in an in vitro hypoxic environment.

Our results showed that Ang-2 and VEGF protein expression levels in BMVECs under an OGD environment were significantly increased, which was consistent with previous studies (Abdulmalek et al. 2001). In addition to being markers reflecting secondary injury, Ang-2 has been shown to cause blood–brain barrier damage and increased endothelial cell apoptosis. The results showed that the injection of Ang-2 in the cerebral cortex can induce significant blood–brain barrier damage in the region surrounding the injection area, as well as leptomeningeal vascular endothelial cell apoptosis. Injection of 400 ng Ang-2 has been shown to produce a relatively large area of extravasation horseradish peroxidase. The study also showed that blood brain barrier damage in the early stages after injury was related with the rapid increase and mobilization of Ang-2 expression and the reduction of Ang-1 levels, the latter being structurally formed and expressed in endothelial cells. Ang-2 expression increases occurred in the first few hours after damage. In the late stages, Ang-2 and activated caspase-3 expression was co-localized in endothelial cells, suggesting that Ang-2 played a role in endogenous-and exogenous-triggered endothelial cell apoptosis (Nag et al. 2011). Our results showed that DHA can reduce apoptosis of BMVECs in an in vitro hypoxic environment, which may be related to its role in reducing Ang-2 generation.

Ang-2 expression level increases under an anoxic environment has been shown to be related to hypoxia-inducible factor 1 (HIF-1), as well as the expression of cyclooxygenase 2 (COX-2) and concomitant PGE2 and PGI2 secretion (Pichiule et al. 2004; Meijerink et al. 2015). COX-2 inhibitors can be used to reduce hypoxia-induced Ang-2 levels, and was closely related with the reduction of PGE2 and PGI2 levels. These studies confirmed that the formation of COX-2-dependent prostaglandins played a more important role in regulating the generation of hypoxia-induced Ang-2. In a colon tumor cell model, it was found that high levels of COX-2 expression promoted the synthesis of angiogenic factors such as VEGF and Ang-2 (Yoshid-Amano et al. 2003). In vivo studies have shown that PGE(2)-stimulates VEGF expression in rat gastric microvascular endothelial cells (RGMEC) via transactivation of JNK1 by ERK2 (Pai et al. 2001). Our results indicated that DHA pretreatment reduced COX-2 expression and prostaglandin production in a dose-dependent manner. COX-2 expression was found to be positively correlated with PGE2 and PGI2 secretion levels. In vivo studies have also shown that DHA can reduce oxidative stress and cerebral ischemia–reperfusion injury by reducing COX-2 expression (Quartu et al. 2012).

Our experiments have also found that DHA pretreatment can reduce the transcription levels of *Ang2* and *Vegf* mRNA. Correlation analysis showed that COX-2 expression was positively correlated with *Ang2* and *VEGF* mRNA levels, and *Ang2* and *Vegf* mRNA levels were positively correlated with Ang-2 and VEGF secretion levels respectively. These results indicated that COX-2 exerted transcriptional regulatory effects on Ang-2 and VEGF in cells via the prostaglandin pathway. Recent studies have found that DHEA (one polyunsaturated fatty acid) can inhibit the production of macrophage inflammatory cytokines through COX-2-mediated transcriptional regulation.

DHA are essential highly unsaturated fatty acids in the body, which regulate a variety of gene transcription targets through binding with PPARs and others (Randy and Guoying 2007). PPARs are members of the nuclear receptor superfamily, which are actively involved in immune regulation by regulating membrane lipid composition, cell proliferation, sensitivity to apoptosis, energy homeostasis, and various inflammatory transcription factors. Although all three PPAR subtypes of are involved in the pathophysiology of brain damage, PPAR-γ has been established as the most closely related with brain injury, and is the most thoroughly studied receptor subtype (Benedusi et al. 2012). In the present study, it was found that the PPAR-γ inhibitor GW9662 can inhibit the protective effects of DHA, further confirming that PPAR-γ is closely related to brain damage.

In this study, the culture media used to treat BMVECs was a sugar-free and serum-free DMEM culture media. BMVECs were placed in a three-gas incubator to create a 94 % N_2_: 5 % CO_2_: 1 % O_2_ hypoxic environment and cultured for 24 h to simulate in vivo ischemia. This model has been used relatively infrequently compared with the hypoxia/reoxygenation model used in most previous studies, and may induce less damage to cells compared with the hypoxic/reoxygenation model.

The following deficiencies may exist in the present study. First, the result that DHA can reduce Ang-2 expression and generation cannot be fully confirmed as the sole mediator of the protective effect, and a specific inhibitor of Ang-2 was not used as a control. Therefore, DHA-mediated suppression of Ang-2 as a protective mechanism may be just an indirect speculation. Second, the present study only used a model of hypoxia, which was also quite different from other hypoxia/reoxygenation models. In addition, in vitro and in vivo models are very different. As such, this phenomenon, as well as the relationship between DHA mechanisms and the angiopoietin pathway regarding cytoprotection, need to be confirmed in future studies performed using in vivo models.

In summary, DHA treatment induced decreases in Ang-2 and VEGF levels under an OGD environment, which may be associated with decreased COX-2 protein expression and PGE2 and PGI2 secretion, as well as the generation regulation by intracellular transcriptional pathways. These findings may have wide therapeutic applications regarding the treatment of acute cerebral ischemic damage and post-secondary damage, and provides some scientific basis for future clinical application.
